# A novel hybrid framework for metabolic pathways prediction based on the graph attention network

**DOI:** 10.1186/s12859-022-04856-y

**Published:** 2022-09-28

**Authors:** Zhihui Yang, Juan Liu, Hayat Ali Shah, Jing Feng

**Affiliations:** 1 School of Computer Science, Wuhan University, Luojia Hill Street, Wuhan, 430072 China; 2 Institute of Artificial Intelligence, Wuhan University, Luojia Hill Street, Wuhan, 430072 China; 3 National Engineering Research Center for Multimedia Software, Luojia Hill Street, Wuhan, 430072 China

**Keywords:** Multi-class classification, Metabolic pathway prediction, Graph attention network, Deep learning, Graph embedding

## Abstract

**Background:**

Making clear what kinds of metabolic pathways a drug compound involves in can help researchers understand how the drug is absorbed, distributed, metabolized, and excreted. The characteristics of a compound such as structure, composition and so on directly determine the metabolic pathways it participates in.

**Methods:**

We developed a novel hybrid framework based on the graph attention network (GAT) to predict the metabolic pathway classes that a compound involves in, named HFGAT, by making use of its global and local characteristics. The framework mainly consists of a two-branch feature extracting layer and a fully connected (FC) layer. In the two-branch feature extracting layer, one branch is responsible to extract global features of the compound; and the other branch introduces a GAT consisting of two graph attention layers to extract local structural features of the compound. Both the global and the local features of the compound are then integrated into the FC layer which outputs the predicted result of metabolic pathway categories that the compound belongs to.

**Results:**

We compared the multi-class classification performance of HFGAT with six other representative methods, including five classic machine learning methods and one graph convolutional network (GCN) based deep learning method, on the benchmark dataset containing 6999 compounds belonging to 11 pathway categories. The results showed that the deep learning-based methods (HFGAT, GCN-based method) outperformed the traditional machine learning methods in the prediction of metabolic pathways and our proposed HFGAT method performed better than the GCN-based method. Moreover, HFGAT achieved higher $F_1$ scores on 8 of 11 classes than the GCN-based method.

**Conclusions:**

Our proposed HFGAT makes use of both the global and local information of the compounds to predict their metabolic pathway categories and has achieved a significant performance. Compared with the GCN model, the introduction of the GAT can help our model pay more attention to substructures of the compound that are useful for the prediction task. The study provided a potential method for drug discovery with all types of metabolic reactions that may be involved in the decomposition and synthesis of pharmaceutical compounds in the organism.

## Background

**Fig. 1:**
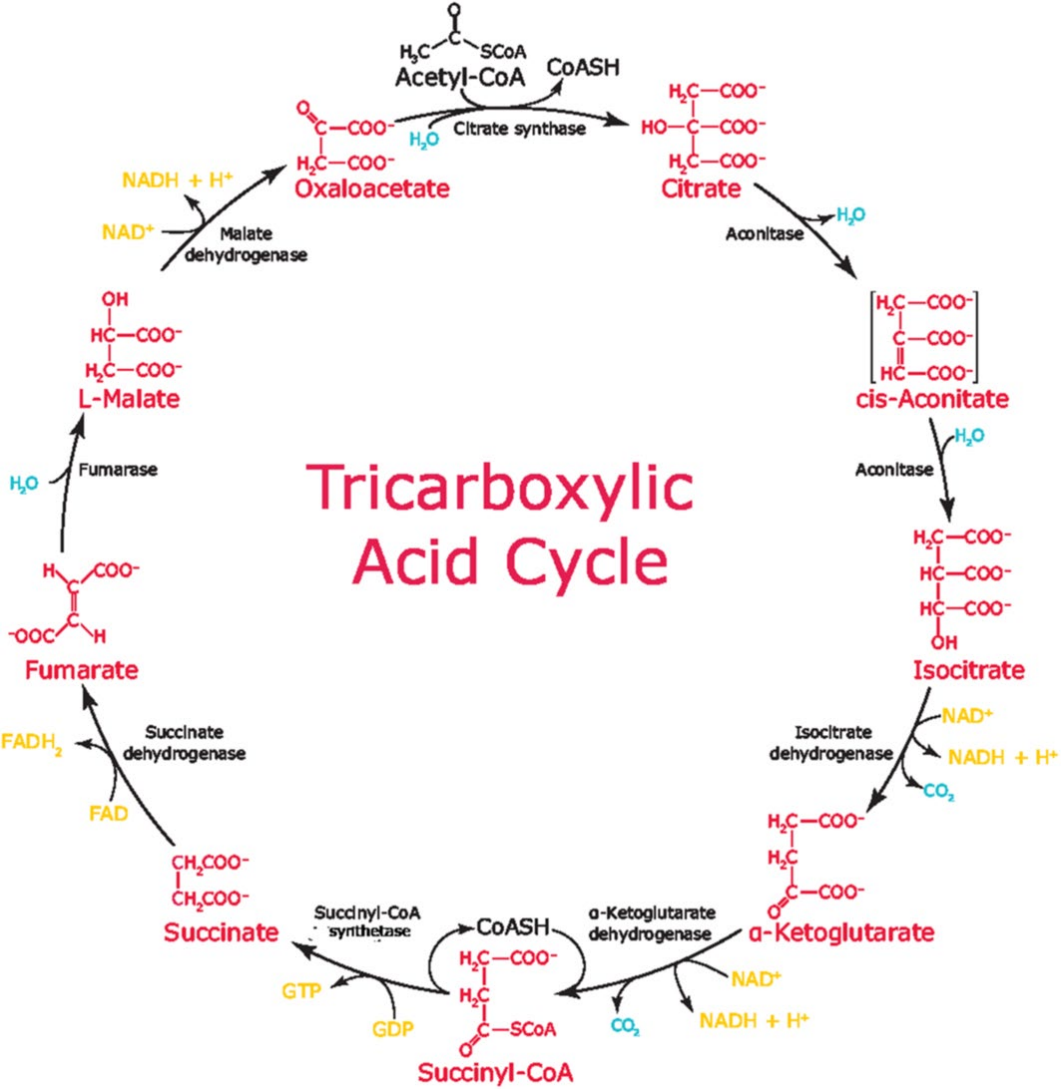
Tricarboxylic Acid Cycle consumes sugars to provide energy for organisms, so all the compounds involves in the TCA cycle are parts of Carbohydrate metabolism category [10]

According to “The Drug Development Process” released by the US Food &Drug Administration, a great number of experiments are designed for ensuring the beneficial effects of a drug molecular compound in the first step of drug discovery and development [1]. The essence of life is metabolism, via which the organism maintains life through a series of biochemical reactions in the body. These reactions participate in different metabolic pathways according to their functions. Therefore, knowing which metabolic pathways that the molecular compounds in a drug are involved can help researchers understand how the drug is absorbed, distributed, metabolized, and excreted [2, 3]. Specifically, a metabolic pathway is a series of biochemical reactions catalyzed by enzymes in cells, which form metabolites to use, store and trigger another metabolic pathway [4]. Different compounds belonging to the same metabolic pathway have similar functions. For example, the main function of the Tricarboxylic Acid Cycle pathway (TCA cycle, KEGG ID:map00020), as shown in Fig. 1, is to provide energy for life activities through the aerobic glucose metabolism [5], meaning that all compounds involves in this pathway play roles in providing energy. Due to the complexity of biological systems, the same compound may also belong to different metabolic pathways and participate in different functions. According to the functional mechanisms, metabolic pathways have been classified into eleven categories [6], such as Carbohydrate Metabolism, Energy Metabolism, Lipid Metabolism, Nucleotide Metabolism and so on. Specific classes of metabolic pathways provide specific roles to the organism. And the main metabolic patterns of different compounds involves in the same type of pathways are similar [7], therefore it is possible to find the potential metabolic process of a drug by identifying the metabolic pathway categories of its compounds. In drug discovery, predicting the metabolic pathway categories of a compound can help to find new drug metabolism and toxic metabolites, thereby reducing the elimination rate of candidate drugs [8, 9].

With the accumulation of experimental data and the development of machine learning techniques, many researchers have paid more attention to building machine learning based on computational models for identifying the metabolic pathway categories that the query compounds belong to. For example, Cai *et al.* built a prediction model based on the KNN (K Nearest Neighbor) method to map small chemical molecules on the metabolic pathways that they may belong to and achieved the accuracy of 77.12% [11, 12]. Hu *et al.* made use of additional information about chemical-chemical interactions to build a multi-target predictor and obtained an accuracy of 77.97% on the dataset of 3137 compounds [13]. Gao *et al.* proposed a hybrid network method which can integrate the information of chemical-protein interactions and protein-protein interactions, as well as the chemical-chemical interactions, and achieved an accuracy of 79.56% on a dataset with 3348 small molecules and 654 enzymes [14].

With the great success of deep learning technologies, many deep neural networks, such as convolutional neural networks (CNNs), graph convolutional networks (GCNs) and recurrent neural networks (RNNs) have been successfully applied in the bioinformatics community  [15]. However, there is little work to focus on building models to predict metabolic pathways that a compound involves in. Recently, Baranwal *et al.* proposed a GCN-based deep learning architecture for metabolic pathway prediction  [16]. Their experiment results showed that the models with the GCN-embedding vector as the features achieved classification accuracies better than competing methods, illustrating the great potential of deep learning-based methods for metabolic pathway prediction. Furthermore, their results also demonstrated the GCN-model with global molecular features outperformed the one without additional features, suggesting that introducing the global molecular descriptors into the deep learning model may help to improve its accuracy.

Inspired by [16], we propose a hybrid framework based on graph attention network (GAT)  [17], aiming to combine both the global and local descriptors of the compounds for the metabolic pathway prediction in this work. Compared with the GCN model, the introduction of the GAT can help our model pay more attention to substructures of the compound that are useful for the prediction task so that the model can achieve higher prediction performance.

## Method

**Fig. 2:**
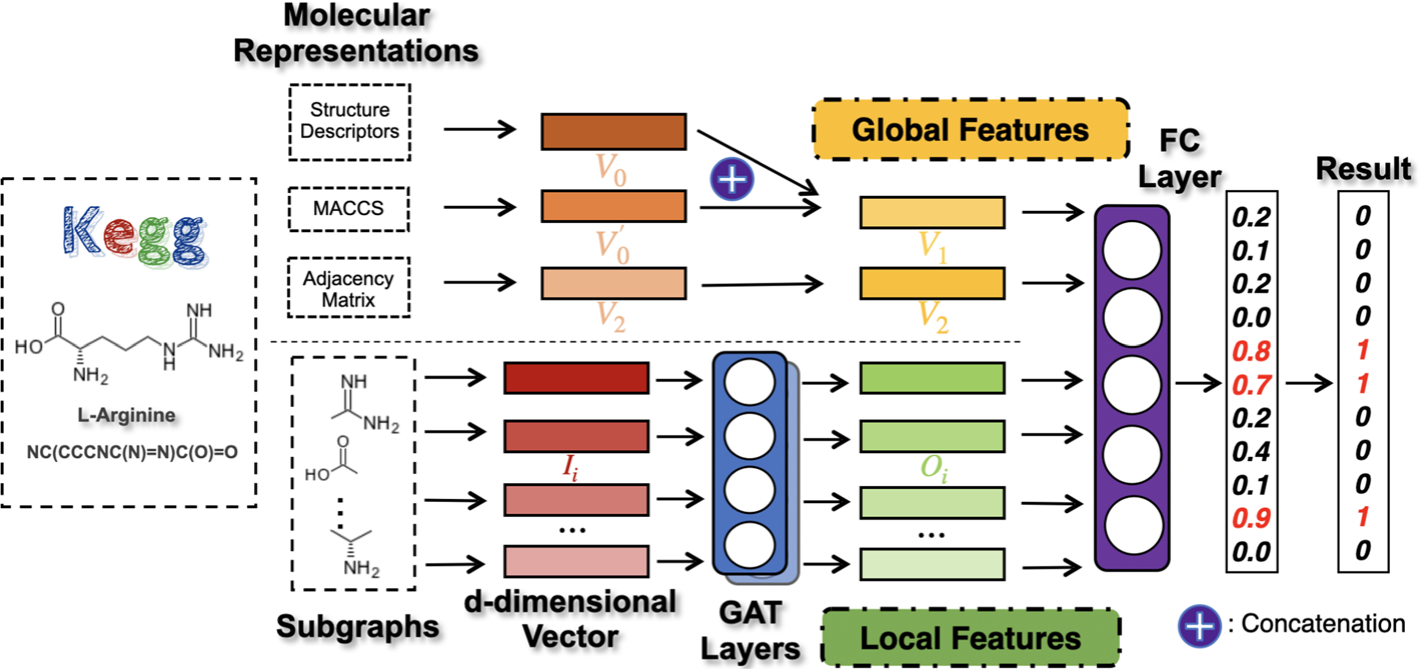
The structure of our hybrid framework

Our proposed hybrid framework based on GAT, named HFGAT, for metabolic pathway prediction is shown in Fig. 2. HFGAT mainly consists of a two-branch feature extracting layer and a fully connected (FC) layer.

In the two-branch feature extracting layer, one branch is responsible to extract global features of the compound; and the other branch introduces a graph attention network (GAT) consisting of two graph attention layers to extract local structural features of the compound. Both the global and the local features of the compound are then integrated into the FC layer which outputs the prediction result of metabolic pathway categories that the compound belongs to. The details of the HFGAT are described in the following.

### Representations of the input compounds

Just like most other researches, we represent the input compound molecules as the SMILES (Simplified Molecular Input Line Entry System) sequences.

The SMILES is a line notation for representing molecules and reactions, which was developed by Arthur Weininger and David in the late 1980s and modified and expanded by other researchers [18]. It is designed for the storage of chemical information and a kind of language with few words (atoms and bonds) and grammar rules. Moreover, this molecular representation is unique and quite compact compared to most other methods (i.e. fingerprint descriptors, molecular map coding and 2D image coding of molecular maps) of representing structure, which makes it the popular representation language of artificial intelligence and chemical experts. For example, the SMILES sequence of L-Arginine is NC(CCCNC(N)=N)C(O)=O.

Every SMILES sequence of a compound molecule can then be processed by using RDKit [19] to generate different kinds of molecular descriptors which can then be used for the subsequent extraction of global and local features of the compound.

### Global features extraction

The top half of the dotted line in Fig. 2 aims for extracting the global features of the compounds. In HFGAT, we extracted two global feature vectors for each compound molecule, denoted as $V_1$ and $V_2$ respectively. $V_1$ mainly characterizes the general molecular information of the compound; $V_2$ mainly describes the connection between the constituent atoms of the compound molecule.

The generation of $V_1$ is as same as  [16]. For completeness, we briefly described the process of generating $V_1$. Firstly, seven molecular descriptors related to the size, rigidity, lipophilicity and polarizability of the compound are extracted to form the 7-dimension vector $V_0$. Each of the descriptor value is obtained by using RDKit [19] . Then the widely used MACCS fingerprint^1^ of the compound is obtained by using RDKit [19] to form the vector $V_0^{'}$. Now that a MACCS fingerprint is a 166-bit binary string where the “1” or “0” respectively indicates the presence or absence of a specific type of substructure in the molecule, $V_0^{'}$ is thus a 166-dimensional binary vector describing the whole strucutral information of a compound. Finally, $V_0$ and $V_{0}^{'}$ are concatenated together to generate the 173-dimensional feature vector $V_1$.

In addition to $V_1$, we also extracted the connection information between atoms of the compound in this work. Concretely, we first generated the adjacency matrix of the compound, in which a row (column) corresponds to an atom of the compound. If there is the connection between atoms *i* and *j*, then the element of (*i*, *j*) is set to “1”, otherwise, “0”. Then we flatten the adjacency matrix as the feature vector $V_2$.

Both $V_1$ and $V_2$ characterize the global information of the compound molecule, thus they act as the global features of the compound.

### Local features extraction

The bottom half of the dotted line in Fig. 2 aims for extracting the local features of the compounds. First of all, a set of subgraphs are extracted from the molecule; then the subgraphs are initially encoded with a set of *d*-dimensional (*d*=70 in this work) random vectors {$I_1$, $I_2$,$\cdots$,$I_n$} (*n* is the number of the atoms of the compound) which are processed by the GAT layers to generate the final feature vectors. Obviously, the GAT helps to embed the local structural information of the subgraphs into the vectors. In this way, a set of feature vectors {$O_1$, $O_2$,$\cdots$,$O_n$} characterizing the local structural information of the molecule can be obtained.

### GAT-based graph embedding

In our work, every local feature corresponds to a subgraph of an atom in the molecule. The subgraph of an atom consists of the atom and all its neighbors, as well as all bonds between the atom and its neighbors.

Just like  [16], we adopted the graph embedding method to obtain the embedding vector for each subgraph, rather than explicitly using the atom and bond features, for the embedding method can easily covert the molecular graph into a low-dimensional vector which requires less computing power to process than the vector in the original form [20]. Different with  [16], we first assigned a *d*-dimensional random vector to every atom and then embedded the structural information of the subgraph of the atom into the vector by using GAT [17]. The key idea of GAT is to introduce the attention mechanism to calculate the influence weight of each node’s neighbors on it, to obtain the overall information of the whole graph from the local information. Therefore, the set of local features obtained from GAT should be beneficial for metabolic pathway prediction.

**Fig. 3:**
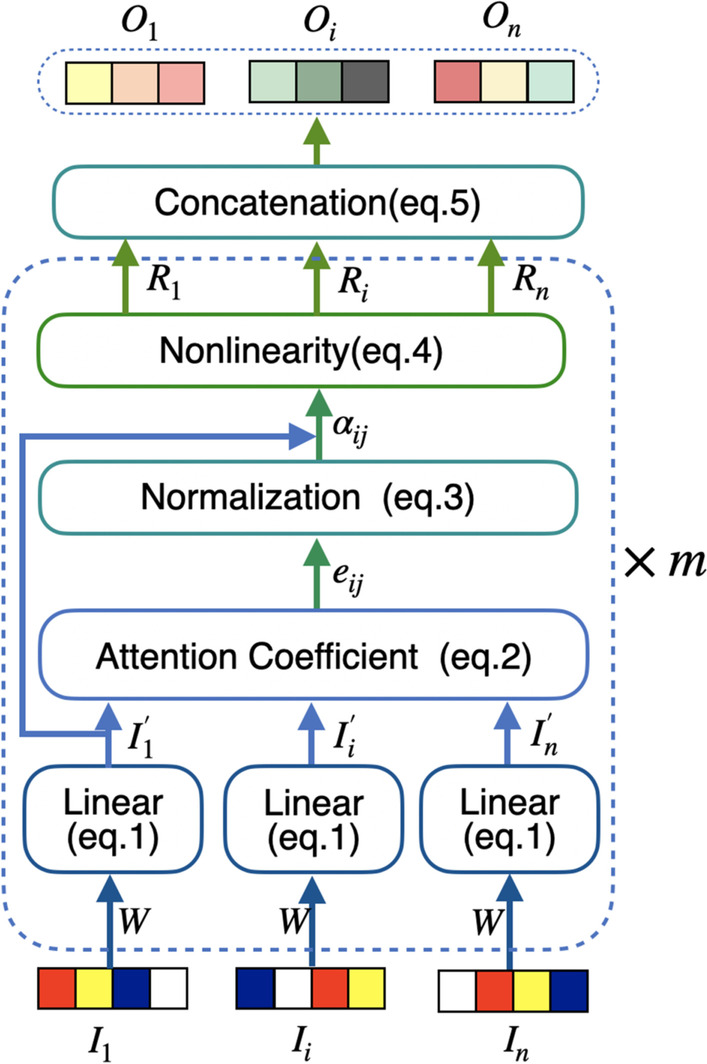
Process of GAT based graph embedding

The process of GAT-based graph embedding is shown in Fig. 3. Every input vector is first linearly transformed by a shared weight matrix $\mathbf {W}$ (parameters to be learnt). Then for each atom, the importance score of each of neighbor atoms of it is calculated by self-attention mechanism and normalized. Finally, all of the normalized scores are used to calculate the nonlinear combination of corresponding feature vectors as the final output feature vector.

In order to make the final output features have powerful representation ability, it is necessary to conduct at least one learnable linear transformation on the input features. Thus a shared weight matrix $\mathbf {W}$ is used to do the linear transformation for every *d*-dimensional input vector $I_i$:

1 $$\begin{aligned} I^{'}_i=\mathbf {W} I_i \end{aligned}$$

where the parameter $\mathbf {W}$ represents the $d \times d$ dimensional weight matrix that will be learnt during the model training process; and it is randomly initialized in the beginning.

#### Calculation of attention coefficient

The self-attention mechanism based on parameterized *LeakyReLU* nolinear function is adopted to compute the attention coefficient score between atom *i* and its neighbor atom *j* in GAT [17] :

2 $$\begin{aligned} e_{ij}= LeakyReLU\left( A^{T}\left[ I^{'}_{i} \Vert I^{'}_{j}\right] \right) \end{aligned}$$

where the symbol $'||'$ denotes the operation of concatenation; the parameter *A* represents a 2*d*-dimensional weight vector and is randomly initialized; $j \in \mathcal {N}_i$; and $\mathcal {N}_i$ is the set of neighbor atom nodes of *i*.

The $e_{ij}$ reflects the importance of neighbor atom *j* to atom *i* so that the contribution of *j* to the feature vector of *i* can be accordingly weighted by $e_{ij}$. In order to make the coefficients comparable among different atom nodes, the *softmax* function is introduced to normalize $e_{ij}$ for all *j*:

3 $$\begin{aligned} \alpha _{i j}=softmax _{j}\left( e_{i j}\right) =\frac{\exp \left( e_{i j}\right) }{\sum _{k \in \mathcal {N}_{i}} \exp \left( e_{i k}\right) } \end{aligned}$$

#### Generation of the feature vector

After obtaining the normalized attention coefficients of all *i*’s neighbor atoms, the embedded feature vector of atom *i* can be obtained by the following nonlinear weighted combination:

4 $$\begin{aligned} R_{i}=\sigma \left( \sum _{j \in \mathcal {N}_{i}} \alpha _{ij} I^{'}_{j}\right) \end{aligned}$$

where $\sigma$ is the ELU activation function in GAT  [17]. ELU is also know as Exponential Linear Unit. It can reduce the effect of bias shift and make the normal gradient closer to the unit natural gradient, thus accelerating the learning of the mean towards zero.

Since it has been found that the multi-head attention by independently executing the above procedure more than once and concatenating the outputs as the final feature representation is beneficial to stabilize the learning process of self-attention in GAT [17], we also adopted such strategy in this work. In particular, different heads pay attention to different positions of the graph structure, thus the extracted features are different and complement with each other. Suppose the transformation process is independently executed for *m* times (*m*=2 in this work) and the output of Eq. (4) is $R^{k}_{i}$ for atom *i* in the $k\hbox {th}$ execution, then we can get the final embedded feature vector $O_{i}$ characterizing the subgraph centered on atom *i* is the following:

5 $$\begin{aligned} O_{i}=\Vert _{k=1}^{m} R^{k}_{i} \end{aligned}$$

### Fully connected layer

Both the global features ($V_1$, $V_2$) and the local features ($O_1$, $O_2$,$\cdots$) of the compound molecules are input into the FC layer with the SoftMax function for predicting the metabolic pathways. Therefore, the number of the input nodes in the FC layer is the number of the feature vectors, and the number of the output nodes in the FC layer is the number of categories of the metabolic pathways. Since the function outputs a set of probabilities that the compound belongs to specific metabolic pathway categories, we simply use 0.5 as the threshold to make the final prediction. That is, if the probability of a compound belonging to a certain type of metabolic pathway is greater than 0.5, then it is considered to belong to the metabolic pathway category.

## Experiment

### Dataset

For the convenience of comparison, we used the same dataset as [16] to do the evaluation experiments. This dataset was collected from the most publicly used biology pathway database KEGG  [6]. Concretely, the dataset contains a total of 6669 compounds which belongs to one or several of 11 manually curated pathway maps that represent molecular interaction and reaction networks. Specifically, 4545 compounds belong to only one metabolic pathway class and the rest belong to more than one metabolic pathway classes. The outline of the dataset is shown in Table 1, in which, “Compound Number” means the number of compounds that belong to the specific pathway class.

**Table 1:** Outline of the dataset*

No.	Metabolic pathway class	Compound number
1	Carbohydrate	1140
2	Energy	768
3	Lipid	1080
4	Mucleotide	356
5	Amino Acid	1454
6	Other Amino Acids	612
7	Glycan	339
8	Cofactors and Vitamins	964
9	Terpenoids and Polyketides	1497
10	Other Secondary Metabolites	1920
11	Xenobiotics	1466

$^*$The dataset was collected by [16]

### Experiments

If a compound belongs to more than one pathway class, only predicting all possible classes at the same time can provide the complete biological functions of the compound. Therefore, we only consider the problem of multi-class prediction in the experiments. In order to evaluate the performance of our methods, we compared HFGAT with other six methods, including five most-used traditional machine learning methods (SVM (Support Vector Machine) [21], *k*NN (*k* nearest neighbor) [11], NB (Naive Bayes) [22], DT (Decision Tree) [23], RF model [24]) and the most recent GCN-based deep learning method GCN+global features [16].

All five traditional machine learning methods were implemented in the Sklearn toolkit [25]; GCN+gloal features methods were implemented in  [16]; Our HFGAT was implemented in Python 3.7.3 on the operation system 64-bit Ubuntu 16.04.6. The parameter settings for all methods are listed in Table 2. For learning algorithms that can only do binary classification, such as SVM, the one-*vs*-one strategy was used for the multi-class classification. The global molecular features were used in all five traditional methods.

**Table 2:** Parameter settings of the methods

Methods	Parameter settings
SVM	Gaussian kernel
*k*NN	*k*=5
NB	Multinomial NB; Laplace smoothing ($\alpha$=1)
DT	Gini impurity; Minimal samples=2
RF	Trees=300; Gini impurity; Depth=60
GCN*	Same as [16]
HFGAT	*d*=70; *m*=2; batch=10; iteration=100

$^*$GCN represents GCN+global features in [16]

We adopted the independent test method to evaluate the methods. Of all 6999 compounds, we random shuffled and spilt them into three sets: the training set (80%, 5335), the validation set (10%, 667 instances), and the test set (10%, 667 instances). All methods run on a GPU server with 4 *NVIDIA GeForce TITAN XP* and 48GB memory.

### Evaluation metrics

We used four metrics to evaluate the performance of the methods: accuracy, precision, recall, and $F_1$. For a binary classifier, these metrics can be calculated by using TP (true positive, the number of positive samples being correctly classified), TN (true negative, the number of negative samples being correctly classified), FP (false positive, the number of negative samples being incorrectly classified as positive) and FN (false negative, the number of positive samples being incorrectly classified as negative). Since we focused on the multi-class classification task in this paper, we followed the idea of  [16] to redefine TP, TN, FP, and FN in terms of the number of correctly (incorrectly) identified classes of a single compound. For example, a compound in the test set is associated with 6 out of 11 pathway categories and its target (true) class labels are represented as the bit-string “10010110101”, where “1” in the *i*th position stands for the compound belongs to the *i*th pathway class, while “0” in the *j*th position indicates that the compound does not belong to the *i*th pathway class. Assume the predicted bit-string of a classifier for this compound is “10101101101”, the TP, TN, FP and FN of this compound are 4, 2, 3 and 2 respectively.

Suppose $TP_i$, $TN_i$, $FP_i$ and $FN_i$ correspond to the *i*th compound, and the number of all compounds is *N*, then we calculate the metrics according to the following equations:

6 $$\begin{aligned} Precision=\frac{1}{N}\sum _{i=1}^{N}\frac{TP_i}{TP_i+FP_i} \end{aligned}$$

7 $$\begin{aligned} Recall=\frac{1}{N}\sum _{i=1}^{N}\frac{TP_i}{TP_i+FN_i} \end{aligned}$$

8 $$\begin{aligned} F_{1}=\frac{2\times {Precision}\times {Recall}}{Precision+Recall} \end{aligned}$$

9 $$\begin{aligned} Accuracy = \frac{1}{N}\sum _{i=1}^{N} \frac{(TP_i+TN_i)}{11} \end{aligned}$$

 Obviously, the precision score measures the average proportion of correctly predicted classes among the predicted classes of a compound. The recall score measures the average proportion of correctly predicted classes in all classes of a compound. The $F_1$ considers the influence of both precision and recall. If one of them is too small, the value of $F_1$ will be smaller. The accuracy score evaluates the average fraction of all correctly predicted associations between compounds and pathway classes.

## Results

**Table 3:** Comparison results of different methods*

Method	Accuracy (%)	Precision (%)	Recall (%)	$\mathbf {F_1 (\%)}$
SVM	90.21±0.13	61.04±0.21	51.87±1.40	56.08±1.26
*k*NN	90.96±0.81	59.61±3.20	62.15±2.80	60.85±1.28
NB	81.97±0.61	45.06±1.60	59.76±1.50	51.37±0.88
DT	81.97±0.61	45.06±1.60	84.56±1.50	81.48±0.88
RF	**97.89±0.12**	84.76±0.78	84.45±0.68	84.60±0.28
GCN	97.61±0.12	89.19±0.52	93.38±0.44	91.17±0.19
HFGAT(*ours*)	97.19±0.06	**90.04±0.28**	**94.12±0.16**	**91.97±0.10**

$^*$The best results are highlighted in bold. GCN represents GCN+global features in [16]. The values before and after the symbol $'\pm '$ respectively represent the mean and standard deviation values

The comparing results of seven methods are listed in Table 3. We noticed that two deep learning-based methods (GCN and HFGAT) reached higher scores on three of four metrics than the five classic methods (SVM, *k*NN, NB, DT, RF). This may be attributed to the representation learning ability of deep learning methods, so they can obtain features more suitable for classification. Combining the learnt features with the handicraft features, GCN and HFGAT can therefore achieve better performance than the traditional machine learning methods. Among the classic methods, RF performed the best. This may be due to the fact that RF is a kind of ensemble classifier so that it can integrate the classification results of multiple classifiers to get better results than a single classifier. We also noticed that RF achieved a slightly higher accuracy score while lower precision, recall and $F_1$ scores than two deep learning-based methods. This would happen when there are many true negatives yet few true positives. Therefore, the accuracy metric alone can not properly evaluate the classification methods.

In Table 3 we also noticed that our HFGAT performed better than the GCN-based method in terms of precision, recall and $F_1$ scores, demonstrating that the use of GAT in HFGAT is helpful for embedding the substructures of the molecular into the local features and benefit for the metabolic pathway prediction.

**Fig. 4:**
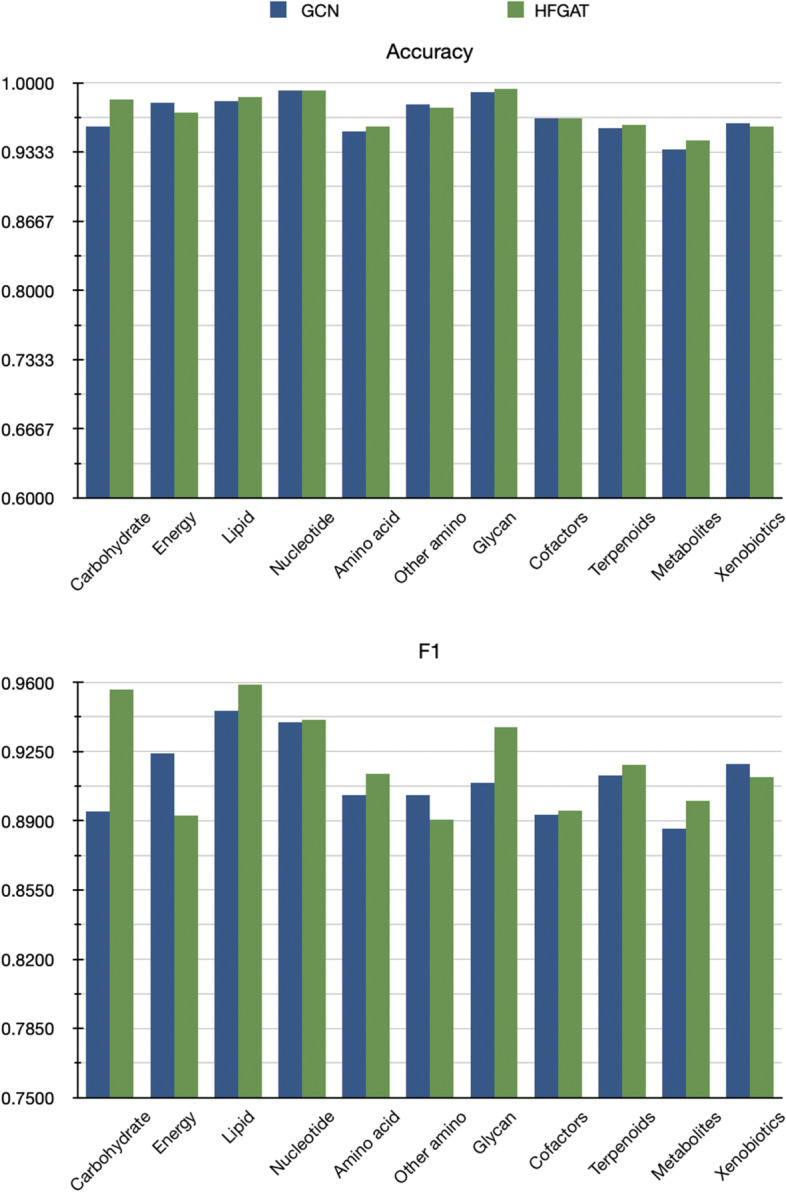
The comparison results between HFGAT and GCN-based method

**Fig. 5:**
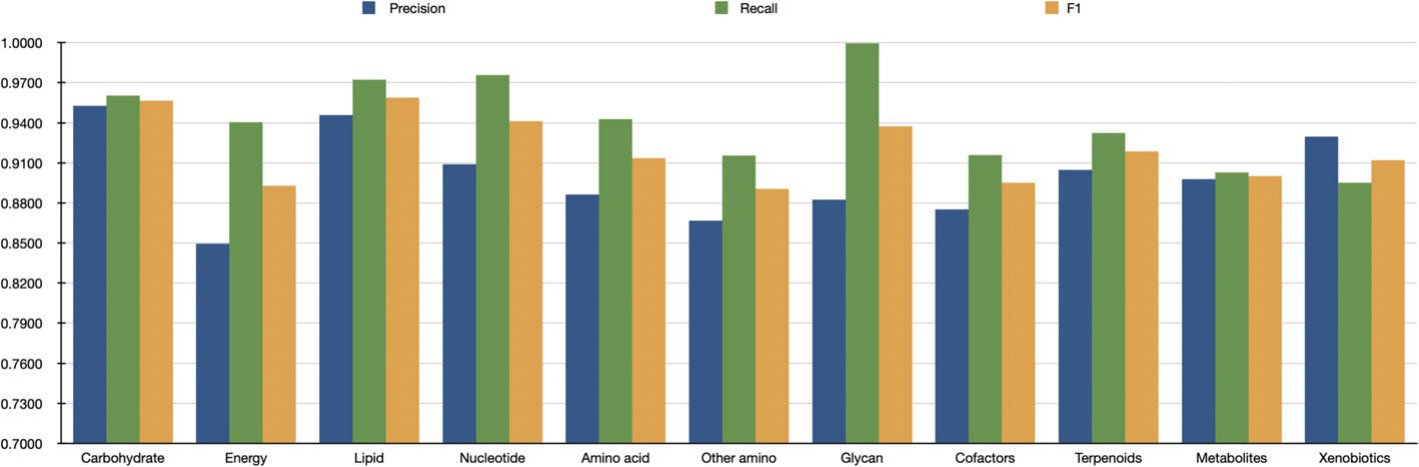
The prediction results of the hybrid framework on eleven classes on KEGG

Table 3 has shown that the overall performance of HFGAT is superior to the GCN-based deep learning method. Since there are 11 pathway classes in the benchmark dataset, in order to know on what classes HFGAT outperforms GCN-based method, we further compared the performance of the two methods to predict different classes of pathways. It should be noted that when calculating the metric scores for a specific class according to the above equations, we only took the compounds belonging to that class into consideration. Without confusion, we still use the same notations. The comparing results of two deep learning-based methods are shown in Fig. 4. We can see that there is little difference between accuracies of the two methods on 11 classes, however, on 8 of 11 classes, the $F_1$ scores of HFGAT are higher than the GCN-based method. The results of HFGAT consistently performing better than the GAT-based method on most classes demonstrate once again that HFGAT can learn more essential substructure features beneficial for pathway classification than the GCN-based method.

## Conclusions and discussion

In this paper, we have presented a hybrid framework based on GAT for the multi-class classification of metabolic pathways, named HFGAT. HFGAT contains a two-branch feature extraction layer, where one branch is used to extract two global molecular feature vectors *V*1 and *V*2, and the other branch is to embed the local structures of the molecular into a set of local feature vectors *O*1, *O*2, ...based on the GAT. The FC layer in HFGAT makes use of both the global and the local feature vectors to predict the metabolic pathways. By comparing five classic methods and one state-of-the-art method on the benchmark dataset, HFGAT has achieved the best performance. The experiment results have shown that (1) deep learning-based methods (GCN-based method and HFGAT) are superior to the classic methods, suggesting the representation learning ability of deep learning can help to obtain suitable features benefit for the classification; (2) the graph attention network used in HFGAT is useful to embed the substructure of the molecular into the local features thus can help to improve the classification performance.

Of course, the local feature extraction method proposed in this paper needs to be improved to fully characterize the local structure of molecules. In fact, by further investigating the precision and recall scores of HFGAT on different classes, shown in Fig. 5, we found that the precision score was generally lower than the recall score on 10 of 11 classes, which demonstrates that the ability of HFGAT to precisely predict the pathway classes of the compounds still needs to be improved, which means that more powerful features may need to be extracted from the molecular.

Firstly, in this work, we only considered the subgraph of each atom and its immediate neighbor. Using atoms and the neighbor nodes with a path length of 2 or more as subgraphs, or using structural units of molecules as subgraphs, whether we can obtain more useful features for classification is a problem worthy of further study. Secondly, when embedding the subgraph into a feature vector, the differences of different bond types between the central atom and its neighbors were not considered in this work. In the graph embedding operation, it is also worth studying to give different weights to different bonds in future work. Finally, we noticed that HFGAT is more complicated than the GCN-based method resulting that it needs much more time to train. Sophisticated optimization skills should also be considered to speed up the training process of HFGAT in the near future.

## Data Availability

The data and materials are available from the corresponding author (Juan Liu: liujuan@whu.edu.cn) on reasonable request.

## Abbreviations

FC: Fully Connected
GAT: Graph attention network
KEGG: Kyoto Encyclopedia of Genes and Genome
RF: Random Forest
GCN: Graph Convolutional Network
TCA: Tricarboxylic Acid Cycle
ML: Machine Learning
KNN: K-Nearest Neighbor
CNN: Convolutional Neural Network
LSTM: Long Short-Term Memory
MACCS: Molecular ACCess System
SMILES: Simplified Molecular-Input Line-Entry System
CART: Classification And Regression Tree
SVM: Support Vector Machine
DT: Decision Tree
GBM: Gradient Boosting Machine.

## Footnotes

1 MACCS was developed by MDL Information Systems, Inc., 14600 Catalina Street, San Leandro, CA 94577. Each MACCS key is a 166-bit binary string, acting as a fingerprint of a compound molecule to describe its structural composition.
